# Infections Caused by *Mycobacterium tuberculosis* in Recipients of Hematopoietic Stem Cell Transplantation

**DOI:** 10.3389/fonc.2014.00231

**Published:** 2014-08-26

**Authors:** Khalid Ahmed Al-Anazi, Asma Marzouq Al-Jasser, Khalid Alsaleh

**Affiliations:** ^1^Section of Adult Hematology and Oncology, Department of Medicine, College of Medicine, King Khalid University Hospital, King Saud University, Riyadh, Saudi Arabia; ^2^Central Regional Laboratory, Ministry of Health, Riyadh, Saudi Arabia

**Keywords:** *Mycobacterium tuberculosis*, hematological malignancy, latency, hematopoietic stem cell transplantation, drug resistance

## Abstract

*Mycobacterium tuberculosis* (*M. tuberculosis*) infections are uncommon in recipients of hematopoietic stem cell transplantation. These infections are 10–40 times commoner in recipients of stem cell transplantation than in the general population but they are 10 times less in stem cell transplantation recipients compared to solid organ transplant recipients. The incidence of *M. tuberculosis* infections in recipients of allogeneic stem cell transplantation ranges between <1 and 16% and varies considerably according to the type of transplant and the geographical location. Approximately 80% of *M. tuberculosis* infections in stem cell transplant recipients have been reported in patients receiving allografts. Several risk factors predispose to *M. tuberculosis* infections in recipients of hematopoietic stem cell transplantation and these are related to the underlying medical condition and its treatment, the pre-transplant conditioning therapies in addition to the transplant procedure and its own complications. These infections can develop as early as day 11 and as late as day 3337 post-transplant. The course may become rapidly progressive and the patient may develop life-threatening complications. The diagnosis of *M. tuberculosis* infections in stem cell transplant recipients is usually made on clinical grounds, cultures obtained from clinical specimens, tissues biopsies in addition to serology and molecular tests. Unfortunately, a definitive diagnosis of *M. tuberculosis* infections in these patients may occasionally be difficult to be established. However, *M. tuberculosis* infections in transplant recipients usually respond well to treatment with anti-tuberculosis agents provided the diagnosis is made early. A high index of suspicion should be maintained in recipients of stem cell transplantation living in endemic areas and presenting with compatible clinical and radiological manifestations. High mortality rates are associated with infections caused by multidrug-resistant strains, miliary or disseminated infections, and delayed initiation of therapy. In recipients of hematopoietic stem cell transplantation, isoniazid prophylaxis has specific indications and bacillus Calmette-Guerin vaccination is contraindicated as it may lead to disseminated infection. The finding that *M. tuberculosis* may maintain long-term intracellular viability in human bone marrow-derived mesenchymal stem cells complicates the development of effective vaccines and strategies to eliminate tuberculosis. However, the introduction of linezolid, cellular immunotherapy, and immunomodulation in addition to autologous mesenchymal stem cell transplantation will ultimately have a positive impact on the overall management of infections caused by *M. tuberculosis*.

## Introduction

*Mycobacterium tuberculosis* (*M. tuberculosis*) is an aerobic, acid fast, non-spore forming non-motile bacillus that belongs to the family *Tuberculosisceae* (1, 2). *M. tuberculosis* is pathogenic for humans while *Mycobacterium bovis* is usually an animal pathogen (1, 2). Tuberculosis (TB) is caused by members of *M. tuberculosis* complex that include: *M. tuberculosis, M. bovis, M. bovis BCG, M. africanum, M. microti, M. pinipedii, M. caprae*, and *M. mungi* (1, 3).

Once infected, active TB disease develops in 10% of patients, while the remaining individuals enter into latency phase that can reactivate at a later time especially if the immunity of the individual declines (1, 4). Active TB develops in approximately 59% of patients and is predominantly pulmonary in nature. Extra-pulmonary TB occurs in 41% of patients and the clinical manifestations depend on the primary site of involvement. Latent TB infection (LTBI) is not contagious, has no clinical manifestations but can reactivate following reduction in immunity (1, 4, 5). Immunocompromised individuals including cancer patients, transplant recipients, and those receiving immunosuppressive therapies including monoclonal antibodies should be evaluated regularly and treated for LTBI at the time of diagnosis or just before starting immunosuppressive treatment (1, 6).

## *M. tuberculosis* Infections in General

Several risk factors predispose individuals to *M. tuberculosis* infections in the general population and these are included in Table 1 (1, 2, 5, 7). Patients with hematologic malignancies (HM) are at risk of developing *M. tuberculosis* infections. Specific predisposing factors for *M. tuberculosis* infections in this group of patients are shown in Table 2 (1, 5, 7).

**Table 1:** **Risk factors for *M. tuberculosis* infections in the general population**.

(1) Human immunodeficiency virus infection
(2) Diabetes mellitus
(3) Hematologic malignancy and solid tumors
(4) Solid organ and hematopoietic stem cell transplantation
(5) End-stage renal disease
(6) Chronic liver disease
(7) Collagen vascular and autoimmune disorders
(8) Chronic and industrial lung diseases such as silicosis
(9) Tobacco smoking
(10) Alcoholism
(11) Use of illicit drugs
(12) Climate and travel
(13) Malnutrition
(14) Pregnancy
(15) Old age
(16) Imprisonment
(17) Genetic predisposition to *M. tuberculosis* infection
(18) Recent exposure to a patient with contagious tuberculosis

*M. tuberculosis, Mycobacterium tuberculosis*.

**Table 2:** **Risk factors for *M. tuberculosis* infections in patients with HM**.

(1) The primary hematological disorder, particularly at the time of relapse
(2) Corticosteroid therapy
(3) Cytotoxic chemotherapy
(4) Radiotherapy
(5) Other immunosuppressive therapies:
- Monoclonal antibodies such as rituximab and alemtuzumab
- Tyrosine kinase inhibitors such as imatinib
(6) Old age
(7) Presence of other comorbid medical conditions such as diabetes mellitus and malnutrition

*M. tuberculosis, Mycobacterium tuberculosis; HM, hematologic malignancy*.

### The global scene of *M. tuberculosis* infections

Approximately eight million new cases of TB infection are reported annually with the vast majority occurring in developing countries and most of the new cases arise as reactivations of old TB infections. Out of these eight million cases, five million patients receive some treatment and only half a million patients receive short courses of direct observed therapy. Recently, the global rates of TB are rising in Asia, Africa, and Latin America where co-infection with human immunodeficiency virus (HIV) is common (1, 8).

The world health organization (WHO) declared TB a global health emergency in the year 1993. One third of the world population has LTBI and 5–10% of latent forms become active at any time. Also, approximately 95% of TB cases and 98% of deaths related to TB infection occur in poor countries (1, 9). Another problem, which makes management of *M. tuberculosis* infections a difficult task, is the evolution of drug resistance. Resistance to anti-TB chemotherapy can be attributed to: (1) failure to complete the course of anti-TB treatment, (2) weak health-care infrastructures particularly in third world countries, (3) lack of diagnostic techniques and drug susceptibility testing (DST), and (4) having no new anti-TB drugs available since the 1960s till the turn of the century (1, 9). In a survey performed by the WHO in the year 1997, drug resistant TB was found in all 35 countries included in that survey and in the last survey done in the year 2000, drug resistant TB was documented in all 100 countries surveyed (1, 10).

## *M. tuberculosis* Infections in Recipients of HSCT

Hematopoietic stem cell transplantation (HSCT) can be defined as the transfer of HSCs from one individual to another (allogeneic) or the return of previously harvested HSCs to the same individual (autologous) after manipulation of the cells and/or the recipient (11). Despite the recent advances in managing HSCT patients and the progress in supportive care, infection is still reported as the primary cause of death in 8% of autologous HSCT patients and 17–20% of allogeneic HSCT recipients (11). Recipients of allogeneic HSCT have severely impaired cell-mediated immunity as a consequence of: (1) pre-transplant conditioning therapies, (2) immunosuppressive treatments given in the post-transplant period, and (3) graft versus host disease (GVHD) and its treatment, which further suppress the immunity in HSCT recipients. Severe suppression of cellular immunity makes HSCT patients at risk of viral, bacterial, fungal, and mycobacterial infections (12, 13). Recipients of T-cell depleted allografts and those having GVHD may have more extensive and prolonged T-cell immunodeficiency and this consequently predisposes them to severe infections (14). For example, in recipients of T-cell depleted allografts in the United States of America (USA), active TB has been reported in 0.69% of patients and all the reported cases originally came from areas that are endemic for TB (14). At least 25% of *M. tuberculosis* infections in recipients of HSCT result from reactivation of LTBIs (1, 15, 16).

### Risk factors for *M. tuberculosis* infections in HSCT recipients

There are several risk factors for the development of *M. tuberculosis* infections in recipients of HSCT and these are listed in Table 3 (1, 5, 13, 14, 17–27).

**Table 3:** **Risk factors for *M. tuberculosis* infections in recipients of HSCT**.

(1) The primary hematological disorder, particularly:
- Acute myeloid leukemia
- Chronic myeloid leukemia
- Myelodysplastic syndrome
(2) Certain conditioning therapies, particularly:
- Busulphan
- Cyclophosphamide
- Total body irradiation
(3) Corticosteroid therapy
(4) T-cell depletion in allografts
(5) Matched unrelated allogeneic HSCT
(6) Mismatched allografts
(7) Acute and chronic graft versus host disease
(8) Bronchiolitis obliterans
(9) History of *M. tuberculosis* infection

*M. tuberculosis, Mycobacterium tuberculosis; HSCT, hematopoietic stem cell transplantation*.

### Epidemiology of *M. tuberculosis* infections in recipients of HSCT

Infections caused by *M. tuberculosis* are 10–40 times commoner in recipients of HSCT than in the general population. The incidence of *M. tuberculosis* infections in recipients of HSCT varies considerably according to the type of HSCT and to the geographical location (1, 14, 21, 23, 26–30). The prevalence of *M. tuberculosis* infections is higher in recipients of solid organ transplant (SOT) than in HSCT presumably due to the longer immunosuppression in SOT recipients (15, 28, 31, 32). Incidences of *M. tuberculosis* infections in HSCT recipients have been reported to be as low as 0.0014% in the USA and as high as 16% in Pakistan (33). In countries, such as Spain and Turkey, the incidence *M. tuberculosis* was 1.6% while in Taiwan and Hong Kong, it was 8.57%. The vast majority of *M. tuberculosis* infections have been reported from Asia and the incidence of TB in recipients of allogeneic HSCT is directly proportional to the incidence of TB in the general population (1, 14, 21, 23, 26–29). Approximately 95% of TB infections in allogeneic HSCT patients have been reported from developing countries, where HSCT is infrequently performed, while the incidence of *M. tuberculosis* infections in allograft recipients is low in developed countries where HSCT is often performed. Usually, *M. tuberculosis* infections develop 45–365 days post-HSCT and most of the cases reported occurred after 90 days of HSCT. Studies have shown that the diagnosis of TB infections is usually made at a median of 257.2 days after HSCT and the range varies from as early as 21 days to as late as 1410 days post-HSCT (1, 19, 21, 22, 32, 33).

### Clinical aspects of *M. tuberculosis* infections in HSCT recipients

In recipients of HSCT, the lung is the most commonly involved organ and lung involvement has been reported in 50–100% of allografts (1, 21, 33–36). Pulmonary tuberculous infections after HSCT are 13.1-fold higher than in the general population. Also, the incidence of pulmonary TB infections is higher in recipients of allogeneic HSCT than in recipients of auto-grafts. Pulmonary TB may coexist with invasive fungal infections such as mucormycosis or aspergillosis in HSCT recipients (1, 21, 33–36). In these patients, lung involvement by TB may resemble that of invasive fungal pulmonary infections and patients may even present with bacteremia caused by *M. tuberculosis* and a rapidly progressive illness. In such situations, the use of computed axial tomography (CAT scans) and molecular techniques such as polymerase chain reaction (PCR) may accelerate the diagnosis in this group of immunocompromised hosts (34). Pulmonary TB should be considered in the differential diagnosis of lung infections in HSCT recipients living in geographical locations that are endemic for TB (37).

At least one third of *M. tuberculosis* infections in recipients of HSCT are disseminated at presentation with predominant extra-pulmonary involvement. Extra-pulmonary sites of infection include: liver, spleen, bone, bone marrow, brain, and spine (1, 13, 16, 24, 32, 38, 39). Central nervous system involvement may take the form of space occupying lesions in the brain. Abdominal involvement may be in the form of acute abdomen due to abdominal masses causing acute abdominal pain and intestinal obstruction (1, 13, 16, 24, 32, 38, 39). In recipients of allogeneic HSCT, *M. tuberculosis* infections may present in an atypical manner such as pyrexia of unknown origin, non-specific features particularly in patients with extra-pulmonary involvement. Therefore, it is essential not only to have a high index of suspicion but also to apply prompt and appropriate diagnostic tools as well (13, 40).

The approach to LTBIs in recipients of allogeneic HSCT varies considerably from one transplant center to another (14). The diagnosis of LTBI is also difficult in immunocompromised individuals as they are likely to have attenuated response to tuberculin skin test (TST). To prevent reactivation of LTBI in recipients of allogeneic HSCT, it is recommended to consider administration of isoniazid (INH) prior to and post-HSCT particularly in patients living in areas that are endemic for TB (14).

### *M. tuberculosis* infections in recipients of certain types of HSCT

*M. tuberculosis* infections have been reported in various forms of HSCT (5, 16, 19, 26, 29, 32, 41–44). The exact incidence of *M. tuberculosis* infections among recipients of umbilical cord blood transplant (UCBT) is unknown (16, 26, 41). The interval between UCBT and the diagnosis of TB infection varies considerably from as early as 21 days to as late as 3 years post-transplant. Also, *M. tuberculosis* infections in recipients of UCBT may have a rapidly progressive course and present with bacteremia or disseminated infection (16, 26, 41). Infections caused by *M. tuberculosis* have been reported in patients receiving autologous HSCT and the incidence varies from 0.0 to 0.23% (5, 19, 32, 42). Old literature suggested that the risk of *M. tuberculosis* infections in recipients of autologous HSCT was similar to that in the general population and that no obvious risk factors were associated with the development of TB infections in autograft recipients (19, 29). However, over the last decade, *M. tuberculosis* infections have been increasingly reported in recipients of autologous HSCT (43, 44). Also, such infections have been reported as early as day 30 post-autologous HSCT and *M. tuberculosis* infections in these patients have been reported to coexist with other infections such as cytomegalovirus infection and adenovirus-related hemorrhagic cystitis. *M. tuberculosis* infections in autograft recipients may present with pulmonary involvement as well as extra-pulmonary manifestations such as bone marrow involvement with pancytopenia and myeloid maturation arrest (43, 44). The recent use of monoclonal antibodies, such as rituximab, to control the primary underlying disease could have contributed to the recent increase in the incidence of *M. tuberculosis* infections in recipients of autologous HSCT (43, 44).

### Diagnosis of *M. tuberculosis* infections in recipients of HSCT

The diagnosis of *M. tuberculosis* infections in HSCT recipients should be based on: clinical grounds, sputum microscopy and cultures, cultures of pleural and pericardial fluid in addition to bronchoalveolar lavage (BAL) samples, bone marrow cultures, serology, molecular testing, and tissue biopsies (1, 40). Tissue biopsies from liver, spleen, lungs, lymph nodes, and bone marrow may show caseating or non-caseating granulomas (1, 40). The histology may be atypical showing either granuloma in the absence of caseation necrosis or lack of granuloma in the presence of caseous necrosis. The latter can be explained by impaired T-cell function in the early post-HSCT phase (1, 16, 26). Unfortunately, a definitive diagnosis of *M. tuberculosis* infections in HSCT recipients is usually difficult to be established because: immunological defecits may lead to mild and non-specific clinical manifestations and histology does not usually show typical granuloma formation (1, 16, 26). Therefore, a high index of suspicion should be maintained in recipients of HSCT living in endemic areas and presenting with: unexplained fever, cough, pleuritic chest pain, diffuse reticulonodular shadows on chest X-ray, rapidly progressive illness, and disseminated infection (1, 12, 20, 32, 37, 40).

## Treatment of *M. tuberculosis* infections in HSCT recipients

Provided there is no evidence of drug resistant strains of *M. tuberculosis*, the recommended treatment schedule for adults with *M. tuberculosis* infections includes: (1) an induction phase for 2 months composed of the standard first-line anti-TB agents: rifampicin: 600 mg/day, INH: 300 mg/day, pyrazinamide 1600 mg/day, and ethambutol 1200 mg/day, and (2) a maintenance phase for 7–10 months with rifampicin 600 mg/day and INH 400 mg/day (21). Multidrug-resistant TB (MDR-TB) has been reported in recipients of allogeneic HSCT. MDR-TB is more frequent in SOT recipients than in HSCT patients (45). The potential risk of MDR-TB may act as a major obstacle to effective treatment of *M. tuberculosis* infections following HSCT despite appropriate anti-TB medications (46). Early introduction of second-line anti-TB chemotherapy may have a good outcome (45).

### Chemoprophylaxis in recipients of HSCT

Isoniazid prophylaxis has been successfully used to prevent reactivation of old TB infections in recipients of HSCT. However, routine prophylaxis against TB infections in patients with skin test reactivity and a normal chest X-ray should be balanced against the possibility of drug-induced hepatotoxicity in recipients of HSCT (1, 18, 23, 29, 35, 37, 42, 47). Therefore, INH prophylaxis should not be given routinely, but close follow-up and monitoring for reactivation of latent infections is recommended. In geographical locations where TB is prevalent: pre-and post-HSCT follow-up for TB should be taken into consideration, and the use of INH prophylaxis should be seriously considered (1, 18, 23, 29, 35, 37, 42, 47).

Indications for INH prophylaxis in candidates and recipients of HSCT include: (1) exposure to an individual with active, infectious (sputum-smear positive) pulmonary, or laryngeal TB regardless the TST or interferon-Gamma release assay (IGRA), (2) Having a positive TST results, regardless of prior bacillus Calmettte-Guerin (BCG) vaccination without previous treatment and no evidence of active TB disease, and (3) having a positive IGRA result, without previous anti-TB therapy and no evidence of active TB disease (11, 15, 35). Exposure of a candidate or a recipient of HSCT to an active, but non-infectious, patient with extra-pulmonary TB does not require preventive therapy. The value of INH prophylaxis in recipients of HSCT living in countries with high prevalence of TB should be considered at institutional or regional levels. In countries with high prevalence of MDR-TB, single-agent prophylaxis may be ineffective (11, 15, 35). In recipients of HSCT living in countries that are endemic for TB, INH prophylaxis has been shown to be effective in preventing TB in these immunocompromised hosts (5, 28, 48). It is recommended that INH should be administered for at least 6–9 months and that it should be commenced prior to or immediately after completion of conditioning therapy for HSCT (11, 28, 48). INH prophylaxis is well tolerated post-HSCT even with concurrent administration of fluconazole. However, concurrent use of INH with itraconazole is not recommended. The impact of using voriconazole or posaconazole on INH prophylaxis is still unknown (11).

Other prophylactic regimens include rifampicin for 4 months although its use is associated with substantial drug interactions with immunosuppressive agents and other medications. Rifampicin and pyrazinamide combination is not recommended because of significant hepatotoxicity that can be caused by this drug combination (11). BCG vaccination is contraindicated in recipients of HSCT due to the risk of having disseminated BCG infection. Donors who live or originate from countries that are endemic for TB have a risk of active TB infection or LTBI similar to the rest of the population. These donors need to be assessed thoroughly and active TB should be ruled out in them prior to donation of HSCs (11).

### Course and prognosis of *M. tuberculosis* infections in HSCT recipients

In recipients of HSCT having *M. tuberculosis* infections, high mortality rates are encountered in patients with miliary TB and disseminated TB infections (1, 16, 19, 21, 33, 36). Mortality rates are higher in allogeneic HSCT than in autologous HSCT recipients. Mortality rates due to *M. tuberculosis* infections in recipients of HSCT range from 0.0 to 75% and mortality is related to the type of HSCT, the degree of immunosuppression, and depends on how early the diagnosis of *M. tuberculosis* infection is made (1, 16, 19, 21, 33, 36).

Usually, *M. tuberculosis* infections in recipients of HSCT are localized to a certain organ such as lungs or central nervous system (1, 16, 30, 31). Occasionally, the infection is disseminated and the course may be rapidly progressive and the following complications may be encountered: disseminated infection, severe hyperpyrexia, adult respiratory distress syndrome, hypotension, hypoxia, sepsis, multi-organ failure, and death. However, early diagnosis, prompt initiation of appropriate anti-TB chemotherapy and having a localized infection as well as a drug-sensitive strain carry good outcome (1, 16, 30, 31).

## New Insights in the Pathogenesis, Diagnosis, and Management of TB

### Recent findings in the immunopathogenesis of TB granulomas

The immunology of TB is complex and multifaceted. Identifying the immune mechanisms that lead to control of initial infection and prevent reactivation of LTBI is crucial to combat TB (49). One of the main features of the immune response to *M. tuberculosis* is the formation of granulomas (50). Understanding granulomas requires an analysis of the complex interplay of innate and adaptive molecular signals that control the focal accumulation of inflammatory cells and activity of their cellular components (51). Granulomas consist of macrophages that produce cytokines such as interferon-γ, highly differentiated cells such as multinucleated giant cells, epithelial cells, and foamy cells, surrounded by a rim of lymphocytes that include CD4 T-cells (50, 52, 53). Despite that granulomas act to constrain the infection, some bacilli can actually survive inside them in a dormant state for a long time (50, 52–54). Although granuloma formation seems to be primarily a host defense mechanism for containing bacteria, it also shelters bacteria by providing them with a niche in which they can persist in a latent form until an opportunity arises for reactivation and spread. Also, evolution of drug resistance develops during the latency phase (50, 52–54).

For unclear reasons, acid fast bacilli (AFB) will reactivate in 10% of the latently infected individuals, escape the granuloma and spread throughout the body, thus giving rise to clinical disease, and are finally disseminated throughout the environment (50). Studies in mice have shown that production of pro-inflammatory cytokines, growth factors, and leukocyte surface markers by granuloma cells indicates continued processes of activation and deactivation of granuloma inflammatory cells during the progress of LTBI (52).

Understanding the pathophysiology of granulomas is critical for the design of new vaccines and anti-TB drugs. Chemokine receptor analysis of granulomas has revealed an age-dependent heterogeneity of immunological responses, thus chemokines may become potential targets for therapeutic interventions (50–52, 55). It is unrealistic to call TB granuloma as an unsuccessful host defense as it successfully contains the infectious focus in more than 90% of cases. The 10% of individuals who progress to active TB suffer from disturbances in the balance between inflammatory reaction to the infectious agent and immunological defenses of the host (54). Granulomas are protective for dormant AFB, but are capable of having tissue destructive nature by behaving as a tumor rather than an active site of bacterial control (55–57). Granulomas are highly dynamic and are shaped by the pathogen and the immune response elements of the host. To secure transmission to a new host, *M. tuberculosis* has evolved to drive T-cell immunity to the point that necrotizing granulomas leak into the bronchial cavities to facilitate expectoration of AFBs. Complete eradication of AFB in granulomas does not occur since *M. tuberculosis* is able to persist within the granuloma, reactivate, and escape under certain circumstances (57).

### Role of mesenchymal stem cells in the pathogenesis of TB

Despite the generation of robust host immune responses, *M. tuberculosis* can successfully evade host immunity to establish a persistent infection by incompletely understood mechanisms. *M. tuberculosis* suppresses T-lymphocyte responses by recruiting mesenchymal stem cells (MSCs) into the site of infection (58). Recruitment of MSCs to the periphery of granulomas plays a crucial role in the pathogenesis of *M. tuberculosis* infection by confining the organism within the granulomas on one hand and keeping M-TB-specific T-cells at the bay on the other hand (58). Thus, large numbers of MSCs infiltrate into the site of TB infection and position themselves between the harbored pathogen and effector T-cells that target the pathogen. MSCs suppress T-lymphocyte responses by producing nitrous oxide. Therefore, targeting MSCs or nitrous oxide seems a feasible therapeutic intervention for designing new effective therapeutic and preventive strategies against TB (58–60).

*M. tuberculosis* may maintain long-term intracellular viability in a human bone marrow-derived CD271^+^/CD45-(BM-MSC) population *in vitro*. Also, *M. tuberculosis* resides in an equivalent population of BM-MSCs in a mouse model of dormant TB infection (61). Viable *M. tuberculosis* has been detected in CD271^+^/CD45^−^ BM-MSCs isolated from individuals who had successfully completed months of anti-TB drug therapy. Thus, CD271^+^ BM-MSCS may provide a long-term protective intra-cellular niche in the host in which dormant *M. tuberculosis* can reside. *M. tuberculosis* can persist in hostile intra-cellular microenvironments evading immune cells and drug treatment. Finally, BM cellular niche may be important for the maintenance of the non-replicating phase of the *M. tuberculosis* life cycle (61). Glycation of the functional domain of CD271^+^ MSCs, which have been proven to be the protective niche for *M. tuberculosis*, occurs in patients with uncontrolled DM and can modulate the genesis of LTBIs in chronic DM (62).

### New diagnostic tools for tuberculosis

The most common direct method for diagnosing TB worldwide is sputum-smear microscopy, which was developed more than 100 years ago, where bacteria are observed in sputum samples examined under a microscope (63). Acid resistance is one of the main features of mycobacteria that allows for quick identification. Consequently, sputum culture remains the gold standard for the diagnosis of pulmonary TB (63). Liquid media are considered the standard method for the isolation and culture of *M. tuberculosis* as they have a better quality of isolation compared to solid media. The introduction of liquid culture-based techniques was a great improvement for diagnosis, shortening the time to detection to 10–14 days instead of several weeks needed for conventional media (63).

Molecular detection of *M. tuberculosis* continues to change the landscape of TB diagnosis. Because of the slow growth rate of *M. tuberculosis*, conventional methods for its detection based on solid culture media take several weeks to yield results (63). With the purpose of obtaining faster results and earlier diagnosis of *M. tuberculosis* infection, several molecular methods have been introduced and evaluated in numerous studies. These molecular tests are summarized in Table 4 (63–68). *M. tuberculosis*-specific nucleic acid amplification tests (NAATs) are the most frequently used molecular tests for laboratory diagnosis of TB (63). The NAAT tests provide a reliable way of increasing the specificity of diagnosis but sensitivity is too poor to rule out disease particularly in smear-negative disease where clinical diagnosis is equivocal and where the need is greatest (4). For pleural TB and TB meningitis, adenosine deaminase (ADA) tests have high sensitivity but limited specificity. NAATs have high specificity to rule in disease and could be used in conjunction with ADA and interferon-γ to increase sensitivity for ruling out disease (4).

**Table 4:** **It shows new diagnostic tests for *M. tuberculosis***.

Category	Type of test/technology used	Examples
Immunodiagnostics	(1) γ interferon release assays; IGRAs for diagnosis of latent TB infection	- Quantiferon-TB gold in-tube (QFT-GIT) - T-SPOT.TB assay
	(2) Tuberculosis biomarkers	(1) Neutrophil percentage in bronchoalveolar lavage
		(2) Serum prolactin level
		(3) C-reactive protein
		(4) Combined interferon-γ inducible protein-10 and interferon-γ
		(5) Combination of 6 serum micro-RNAs:
		- hsa-miR-378, - hsa-miR-29c
		- hsa-miR483, - hsa-miR-101
		- hsa-miR-22, - hsa-miR-320b
Molecular tests used for detection of *M. tuberculosis* in clinical specimens	(1) Nucleic acid amplification assays (2) Lateral flow assays (3) Line probe assays (4) DNA sequencing techniques	- Accu Probe (Gen-Probe, Inc., San Diego, CA, USA) - INNO-LiPA Mycobacteria (LiPA, Innogenetics, Ghent, Belgium) - Geno Type *Mycobacterium* assay (Hain Diagnosika, Germany) - TB peptide nucleic acid fluorescence *in situ* hybridization (Dako, A/S Glostrup, Denmark) - PCR-restriction fragment length polymorphism analysis (PRA) - DNA microarray or high density oligonucleotide arrays
Detection of drug resistance	- Molecular techniques are used in testing drug susceptibility - Genes involved in drug resistance: - kat G - rrs - inh A - rpsL - rpo B - emb B	(1) Line probe assays: PCR-based reverse-hybridization line probe assay (Inno-LiPA Rif TB test, Innogenetics, NV, Ghent, Belgium) (2) Real-time PCR Xpert MTB assay for detection of rifampicin resistant *M. tuberculosis* strains (3) Array-based technologies: multiplex PCR for detection of genetic mutations involved in drug resistance
New technologies in the pipeline for detection of *M. tuberculosis*	(1) LAM-urine antigen detection using ELISA techniques	
	(2) Use of chromatographic techniques for identification of volatile organic compound as markers in clinical specimens	
	(3) Bead-based methods for detection and identification	
	(4) Simplified smart flow cytometry	
	(5) Broad nucleic acid amplification-mass spectrometry	

*M. tuberculosis, Mycobacterium tuberculosis; PCR, polymerase chain reaction; ELISA, enzyme-linked immunosorbent assay*.

Critical parameters of newly developed enzyme-linked immunosorbent assay (ELISA) have been optimized and the cocktail antigens have specificity and sensitivity of >98% as compared to other commercially available diagnostic tests. The newly developed ELISA that employs a cocktail of secretary proteins of *M. tuberculosis*, is an effective tool that can be used for routine screening and early-stage diagnosis of active TB (69).

Automated liquid culture systems and molecular line probe assays are recommended by the WHO as the gold standard first-line DST. The use of this system has not been cleared by the food and drug administration (FDA) in the USA for DST of second-line drugs and thus most laboratories rely on agar proportion as the reference standard (63). With the purpose of detecting drug resistance in a shorter time, several molecular assays have been introduced and these are listed in Table 4 (63–68). Studies have shown that genetic mutations play a significant role in the evolution of drug resistance. Examples of the genes involved in drug resistance are shown in Table 4 (68). Also, examples of the new technologies for detection of *M. tuberculosis* that are in the pipeline are included in Table 4 (65).

### Tuberculosis biomarkers

Tuberculosis biomarkers have the following potential applications: (1) classifying patients at a single time point as having active TB, LTBI, or no disease, (2) predicting future risk of reactivation, (3) monitoring eradication of LTBI, and (4) predicting end points for clinical trials by serving as surrogate markers of cure following anti-TB chemotherapy or vaccination against TB. Unfortunately, the progress in developing biomarkers for TB infection, in general, has been slow (70). However, a number of biomarkers have been shown to be useful in the diagnosis of pulmonary TB in immunocompromised individuals and these are shown in Table 4 (71–74). Several studies testing new biomarkers and utilizing new technologies such as proteomics, transcriptomics and metabolomics are underway. Hopefully, these trials will ultimately determine a panel of biomarkers that can help not only in establishing the diagnosis but also in the follow-up of TB infections (70).

### IGRAs in the diagnosis of TB

The diagnosis of LTBI is recommended in patients with HM and in recipients of HSCT. New *in vitro* T-cell based IGRAs, which utilize specific *M. tuberculosis* antigens have been introduced into routine practice in recent years (75). TST is the most widely used test for the diagnosis of TB infections and it has been an important and a traditional way for detecting LTBIs for a long time. However, the operational and biological limitations of TST indicate the need for an improved approach to diagnose TB infections. An accurate diagnosis of TB infection is very essential to prevent the progress from LTBI to active TB so that the overall burden of TB disease is diminished. In patients with HM and in recipients of HSCT, T-SPOT.TB may be a more useful screening test for LTBI and active TB than TST (75). IGRAs are diagnostic tools for LTBI and they are not affected by BCG vaccination status. There are two major IGRAs available: (1) quantiferon-TB gold in-tube (QFT-GIT) assay and (2) T-SPOT.TB assay (65, 75–77) (Table 4). IGRAs have sensitivity of more than 95% for diagnosis of LTBI. However, the sensitivity of T-SPOT.TB appears to be higher than that of QFT-GIT or TST (76). In the USA, the 2010 centers for disease control and prevention (CDC) guidelines indicate that IGRAs can be used in stead of TST in all situations in which the CDC recommends TST as an aid in diagnosing *M. tuberculosis* infections. IGRAs are preferred for patients with history of BCG vaccination while TST is preferred for testing children under the age of 5 years (76). The 2013 Canadian TB standards indicate that both TST and IGRAs are acceptable alternatives for LTBI diagnosis, either test can be used for LTBI screening in any of the situations in which testing is indicated with some preferences and exceptions. For serial testing in populations exposed to TB, data are insufficient for interpretation of IGRA conversion and reversion (76). Positive QFT.TB assay results predict development of TB in recipients of HSCT in whom LTBI cannot be detected by TST (77).

### The role of radiology in *M. tuberculosis* infections

Pulmonary infections are an important cause of morbidity and mortality during the course of treatment in patients with HM and in recipients with HSCT (78). In these immunocompromised patients: bacterial, viral, fungal, and mycobacterial organisms may infect the lungs (78). Occasionally, the infectious agents can be recovered but, at times, cultures are negative and invasive procedures such as BAL or transbronchial biopsies are required to determine the etiology of pulmonary infiltrates. Radiological findings may be helpful in predicting the etiology of pulmonary infection and may also help in prompt initiation of appropriate antimicrobial therapy (78).

High resolution (HR) CAT scans of chest are the preferred method for evaluation of a lung infiltrate over plain chest radiography because they are more sensitive in detecting pulmonary infiltrates earlier and they are more capable of better characterization of the infiltrates (79). In patients with HM and in recipients of HSCT, HR-CAT scans have a high degree of sensitivity (>85%), a high negative predictive value (>85%) in detecting pneumonia and a gain of 5 days compared to chest X-rays. Consequently, clinical management is changed based on HR-CAT scan findings (79). CAT scan appearances in pulmonary TB include: air space consolidation; pulmonary nodules; interstitial or septal thickening; cavity formation; tree-in-bud sign; ground glass opacification; pleural effusions, as well as enlarged and may be necrotic lymph nodes (78, 80). In patients with febrile neutropenia, HR-CAT scan of chest is an excellent modality in the diagnostic work-up allowing early detection and characterization of pulmonary abnormalities. In pulmonary TB, HR-CAT scans have 90% sensitivity and 97.02% specificity (80).

### The emerging role of PET scans in TB diagnosis

CAT scans and magnetic resonance imaging (MRI) provide excellent structural resolution for visualization of areas of infection and inflammation, but mainly after becoming so advanced to cause significant anatomical tissue damage (81, 82). Fluorodeoxyglucose positron emission tomography (FDG–PET) scans are highly sensitive in detecting foci of infection early and they fulfill most of the criteria established for molecular imaging. In patients with TB infections, FDG–PET scans can show intense multifocal uptake in involved areas, such as lymph nodes, bones, and lung parenchyma, and they determine active foci of infection as well as the extent of these infections. They are useful in monitoring and follow-up of complications related to these infections (81, 82).

On PET scans, TB appearances may mimic those of malignancy such as lymphoma or other infections such as invasive fungal infections (83). C-11 acetate accumulates in tumors but not in infectious or inflammatory lesions. Therefore, incorporation of C-11 acetate in PET scanning may help to differentiate TB from malignancy (83). Although a high standardized uptake value (SUV) of more than 2.5 is usually attributed to malignancy, SUV values ranging between 2 and 21 have been described in patients with tuberculous infections (83).

### New anti-TB therapies

Barriers to improvements in the outcomes of anti-TB therapies include: (1) long treatment duration resulting in poor patient adherence to treatment and loss of patients follow-up, (2) complex therapeutic regimens that involve expensive and toxic medications, (3) toxic effects and drug interactions particularly if the patient is receiving other medications such as antiretroviral treatment or immunosuppressive therapies following transplantation, and (4) evolution of MDR strains (84). MDR isolates of *M. tuberculosis* arise as a consequence of sequential accumulation of a genetic mutation conferring resistance to single therapeutic agents (85). MDR-TB is caused by *M. tuberculosis* that is resistant to at least INH and rifampicin, which are the two most effective first-line anti-TB drugs (84, 86, 87). Extensively drug-resistant TB (XDR-TB) is MDR-TB with additional resistance to any of the fluoroquinolones and to at least one of three injectable anti-TB agents such as amikacin, kanamycin, and capreomycin (84, 86, 87).

After 50 years of no new anti-TB drug development, a promising pipeline is emerging through the repurposing of old drugs, re-engineering of existing antibacterial compounds, and discovery of new and novel therapies that are active against dormant as well as persistent populations of *M. tuberculosis* (84). New drugs and chemical compounds have been tried with success in the treatment of infections caused by *M. tuberculosis* and these are included in Table 5 (84, 88, 89). New drugs and new combination regimens in clinical trials are expected to increase therapeutic efficacy and shorten treatment duration in both drug-susceptible and drug-resistant strains of *M. tuberculosis* (84, 88, 89). Studies in non-human primate models of active TB and LBTI have shown that targeting mycobacteria in hypoxemic environments by administration of drugs such as metronidazole may not only prevent reactivation of LTBI but may shorten the treatment duration of active TB as well (90).

**Table 5:** **It shows new therapeutics active against *M. tuberculosis***.

Type of therapy	Examples
(1) New antimicrobials active against *M. tuberculosis*	• New fluoroquinolones and fluoroquinolone containing compounds	
	- Levofloxacin	- OFLOTUB
	- Moxifloxacin	- NIRT
	- Rifaquine	- REMoxTB
	• Linezolid,	• Rifapentine
	• Clofazimine,	• Bedaquiline and delamanid
(2) Chemical agents active against *M. tuberculosis*	• Sutezolid,	• OPC-67683
	• AZD 5847,	• SQ-109
	• PA-824,	• BTZ-043
	• TMC-207,	• PNU-100480
(3) Adjunctive immunotherapy	• IL-2,	• IL-24
	• IL-7,	• Interferon-γ
(4) Autologous mesenchymal stem cell transplantation	• In conjunction with anti-tuberculous chemotherapy	
	• Effective against MDRTB and XDRTB strains	
(5) Targeted *M. tuberculosis* therapy	• Animal studies have shown that metronidazole targets *M. tuberculosis* in hypoxemic environment	

*M. tuberculosis, Mycobacterium tuberculosis; IL, interleukin*.

*MDRTB, multidrug-resistant tuberculosis; XDRTB, extensively drug resistant tuberculosis*.

The use of immunotherapy as an adjunct to anti-TB chemotherapy may improve success rates for treatment of MDR-TB, shorten the time for drug-sensitive TB, and improve the immunity of individual by enhancing TB elimination to prevent disease recurrence. The adjunctive immunotherapies are listed in Table 5 (91).

### Role of autologous HSCT in the treatment of *M. tuberculosis* infections

Recently, autologous HSCT has successfully been used in the treatment of MDR-TB and even XDR-TB (Table 5) (92, 93). In one study, 27 patients, in whom previous long-term treatment with anti-TB drugs alone had been ineffective, were included (15 with MDR-TB and 12 with XDR-TB). All patients received autologous MSCs. Positive clinical responses were obtained in all 27 patients, bacterial discharge from lungs stopped in 20 patients after 3–4 months, and resolution of tissue damage and lung cavitation was obtained in 11 patients (92). In the 16 patients who had follow-up for 18–24 months: persistent remission of tuberculous process was achieved in nine patients and significant positive bacteriological and morphological responses were obtained in six patients. So, inclusion of transplantation of autologous MSCs into the course of anti-TB treatment may be a promising maneuver to enhance the efficacy of treatment in patients with drug-resistant pulmonary TB (92).

In another phase 1 clinical trial, 30 patients with microbiologically confirmed MDR-TB or XDR-TB were included (93). After 4 weeks of anti-TB drug therapy autologous HSCT was performed. Autologous bone marrow-derived MSCs were infused and the dose of MSCs was 1 × 10^6^ cells/kg body weight. The adverse events encountered were mostly grade I or II (93). Therefore, in patients with drug resistant TB, autologous MSCs can be used in combination with standard anti-TB chemotherapy. However, adjunct therapy using MSCs needs to be evaluated in controlled phase 2 trials to assess effects on immune responses and clinical as well as microbiological outcomes of this newly evolving therapeutic modality (93).

### Vaccination against *M. tuberculosis* infections

The BCG vaccine was developed as an attenuated live vaccine for TB control almost a century ago. Despite being the most widely used vaccine in human history, it has two major limitations: its poor efficacy against adult pulmonary TB and its disconcerting safety in immunocompromised hosts (94–96). During the past 5 years, an alarming increase in the number of patients with MDR-TB and XDR-TB has been noted, particularly in East Europe, Asia, and South Africa. Treatment outcomes, with the available therapeutic regimens for DR-TB, are usually poor (97). Although substantial progress in drug development for TB has been achieved, scientific progress toward development of interventions for prevention and improvement of drug treatment outcomes have lagged behind. So, innovative interventions and novel adjunct treatments are needed to combat the growing pandemic of MDR-TB and XDR-TB by improving its cure rates (97).

A novel, safe, widely applicable and more effective vaccine against TB is desperately sought to achieve disease control. Over the last 20 years, tremendous progress has been achieved in TB vaccine research and development from a pipeline virtually empty of new TB candidate vaccines in the early 1990s to an era in which almost 20 vaccine candidates are present at different stages in the clinical trial pipeline (97, 98). The potential TB vaccines are either subunit vaccines aimed at boosting BCG-prime vaccination or recombinant BCG constructs that may replace BCG vaccine in the future (95, 96). Additional vaccine candidates will enter clinical trials in the near future including post exposure vaccines for individuals with LTBI. Ultimately, vaccines that prevent or eradicate *M. tuberculosis* infection would be the best possible option (95). The next 10–15 years will be critical for TB vaccines to demonstrate protective efficacy and safely. Next generation vaccines should be designed with the aim of preventing infection or achieving sterile eradication perhaps by redirecting immune responses at *M. tuberculosis* antigens expressed during latency or by using new platforms (98).

Having a strategy of host-directed therapies focused on the immune response of anti-TB treatment could be particularly beneficial for patients with MDR-TB or XDR-TB. Hence, more attention should be given to host-directed preventive and therapeutic interventions (97). Improved knowledge of immunology, molecular microbiology, cell biology, and biotechnology has paved the way toward effective and safe vaccines against TB. The pipeline of new vaccine candidates from preclinical to clinical testing could be accelerated by development of biomarkers that can predict the clinical outcome of TB (95). As there is no clear regulatory pathway for TB vaccines, global sharing of information among regulatory authorities from countries engaged in development of TB vaccines is required (99).

## Conclusion

*M. tuberculosis* infections carry significant morbidity and mortality in recipients of various forms of HSCT particularly in patients living in endemic geographic locations. These infections have a number of risk factors and they cause a wide spectrum of clinical manifestations and complications. A high index of suspicion should be maintained in HSCT recipients presenting with compatible clinical or radiological features. Prompt diagnosis and early institution of appropriate therapy are associated with favorable outcome.

The plethora of new diagnostic techniques will hopefully help clinicians, radiologists, and laboratory staff to have early diagnosis. The newly evolving therapeutics, cellular therapies as well as vaccines will ultimately be translated into more optimal management of these potentially life-threatening infections in this peculiar group of immunocompromised patients.

## Conflict of Interest Statement

The authors declare that the research was conducted in the absence of any commercial or financial relationships that could be construed as a potential conflict of interest.
